# Case series: Effects of a ketogenic diet on cardiometabolic health in seven outpatients with bipolar disorder

**DOI:** 10.3389/fnut.2025.1635489

**Published:** 2025-10-07

**Authors:** Louis Schreel, Maxi Bürkle, Andrea Thiem, Volker von Baehr, Joshua Sauren, Gerrit Keferstein

**Affiliations:** 1 MOJO Institut für Regenerationsmedizin, Hennef, Germany; 2 IMD Labor, Berlin, Germany

**Keywords:** metabolic psychiatry, ketogenic metabolic therapy, bipolar disorder, cardiometabolic health, lipids-metabolism, inflammation, oxidative stress

## Abstract

**Background:**

Recent research suggests that the ketogenic diet (KD) has the potential to serve as an effective treatment option for neuropsychiatric disorders, targeting both dysfunctions in brain metabolism and cardiometabolic comorbidities. In many patients, KDs may ameliorate comorbidities such as obesity, metabolic syndrome and type 2 diabetes. However, the long-term effects of KDs on cardiovascular health remain an important topic of investigation, due to considerable inter-individual variability in how KDs may impact lipid metabolism. While some studies have shown no significant change in cholesterol levels, and some have shown improvements, still others have highlighted the potential of KDs to induce or exacerbate hyperlipidemia.

**Objective:**

To shed new light on this ongoing controversy, we present both beneficial and concerning effects of a 3-month intervention with Ketogenic Metabolic Therapy (KMT) (1.5:1 ratio) on a wide range of cardiometabolic health markers in seven outpatients with bipolar disorder and comorbid dyslipidemia.

**Methods:**

Cardiometabolic assessments were based on markers of lipoprotein burden, such as apolipoprotein B (apoB), lipoprotein (a) (Lp(a)), low-density lipoprotein cholesterol (LDL-C), high-density lipoprotein cholesterol (HDL-C), triglyceride (TG) and total cholesterol (TC), markers of inflammation, such high-sensitivity C-reactive protein (hsCRP) and tumor necrosis factor (TNF-α), markers of oxidative stress, such as malondialdehyde-modified LDL (MDA-LDL) and nitrotyrosine, molecules that relate to endothelial function, such as homocysteine and advanced glycation end products (AGE), and anthropometric measures, such as BMI and fat mass. Mean changes between pre-KMT and post-KMT measurements were calculated. In addition, within-person changes in outcomes of interest were visually summarized using boxplots.

**Results:**

Beneficial cardiometabolic effects included a decrease in mean Lp(a) of 6.6 mg/dl (−21%), a reduction in mean triglyceride of 40.6 mg/dl (−30%), a reduction in mean apoB of 0.14 g/L (−10,5%) and an increase in mean HDL-C of 3 mg/dL (+5%), a reduction in mean hsCRP of 0.94 mg/L (−45%), a reduction in mean TNF-α of 1.31 pg/ml (−7%), a reduction in mean MDA-LDL of 36.77 U/L (−38%), a reduction in mean nitrotyrosine of 225 nmol/L (−28%), a mean weight reduction of 4 kg (−4,6%), a mean visceral fat reduction of 0.69% (−10%) and a mean fat mass reduction of 3.7 kg (−12%). However, some concerning effects were also observed. Of note, mean homocysteine levels increased by 1.94 umol/L (+18%) and mean AGE levels increased by 30.9 ug/ml (+106%). Moreover, mean LDL-C was increased by 14 mg/dl (+9%) and mean total cholesterol was increased by 7 mg/dl (+3%).

**Conclusion:**

Based on these findings, it is concluded that comprehensive Ketogenic Metabolic Therapy provided to outpatients with bipolar disorder can be beneficial in improving a broad range of cardiometabolic health markers, including lipid metabolism, inflammation, oxidative stress and anthropometric measures. Tentatively, these findings suggest that at least a proportion of patients with bipolar disorder may find remarkable improvements in cardiometabolic health adopting a metabolic treatment such as the ketogenic diet. However, potentially concerning effects on markers such as homocysteine and AGE call for well-formulated, individualized KDs.

## 1 Introduction

Compared with the general population, patients with bipolar disorder (BD) have significantly higher rates of cardiometabolic comorbidities, including obesity, metabolic syndrome, dyslipidemia, hypertension, insulin resistance and type 2 diabetes (1–4). These comorbidities place the bipolar population at higher risk for developing cardiovascular disease (CVD) and reduce life expectancy in this population. Studies have indicated that patients with bipolar disorder lose on average 10 years of their normal lifespans, with excess mortality being primarily due to preventable diseases such as cardiovascular disease and type 2 diabetes (5, 6). The most common cause of death in the bipolar population, responsible for about a third of all deaths, is CVD, which often goes underdiagnosed and undertreated (7–9).

The increase of cardiometabolic comorbidities in the bipolar population may be related to both lifestyle habits (e.g., smoking, poor diet, lack of exercise) and medication side effects, which affect the metabolic, hormonal and neurologic systems (8). These side-effects are well established. Lithium can cause weight gain (10, 11) and adversely influence glucose metabolism (12). Second generation antipsychotics such as quetiapine, clozapine, asenapine, lurasidone, etc. are associated with hyperlipidemia (13–17), insulin resistance or increased risk of diabetes mellitus (14, 18–23).

In addition to worsening physical health, cardiometabolic comorbidities also contribute to poorer response to psychiatric treatment, and are associated with more severe mood symptoms (24–26). BD patients with comorbid obesity have been reported to have a more chronic course of illness, more severe symptoms (including risk of suicide), anxiety symptoms, poor cognitive performance and poor response to lithium (24, 27, 28). Similarly, BD patients with comorbid insulin resistance or type 2 diabetes have been reported to have three times higher odds of a chronic course of bipolar disorder compared with euglycaemic (glucose tolerant) patients, three times higher odds of rapid cycling and to be more likely to be refractory to lithium treatment (29).

In recent years, there has been a remarkable surge in interest in the ketogenic diet (KD) as a metabolic intervention for psychiatric diseases, targeting both dysfunctions in brain metabolism and cardiometabolic comorbidities. Although there is only a small number of clinical trials investigating the therapeutic potential for psychiatric disorders, current evidence from case reports (30–32) and pilot studies (33–35) is promising. Implementing Ketogenic Metabolic Therapy (KMT) in patients with mental disorders has been a feasible and well-tolerated approach, resulting in remarkable reductions in psychotic and affective symptoms and significant improvements in metabolic health. More than a dozen clinical trials of KMT for serious mental illness are currently underway for conditions including bipolar disorder, major depression, and schizophrenia spectrum disorder (36–46).

Systematic reviews and meta-analyses of randomized clinical trials (RCTs) have also reported moderate to high-quality evidence for the diet’s efficacy in treating metabolic syndrome, obesity and type 2 diabetes, and in positively affecting problematic cardiometabolic markers (47). However, studies have also shown there is considerable inter-individual variability in the low-density lipoprotein cholesterol (LDL-C) and total cholesterol response to ketogenic dietary interventions. While some studies show no changes in cholesterol levels, and others show improvements, still others have shown that KDs have the potential to induce or exacerbate hypercholesterolemia, raising both total cholesterol (TC) and LDL-C (48). This is particularly significant for patients in psychiatric care, given their increased risk of cardiometabolic comorbidity.

The objective of this retrospective study is to display both potentially beneficial and potentially harmful effects of the ketogenic diet on a broad spectrum of cardiometabolic health markers in bipolar patients with underlying hyperlipidemia. We evaluate cardiometabolic health effects based on markers of lipoprotein burden, such as apolipoprotein B (apoB), lipoprotein (a) (Lp(a)), triglyceride (TG), total cholesterol (TC), high-density lipoprotein cholesterol (HDL-C) and low-density lipoprotein cholesterol (LDL-C), measures of inflammation, such high-sensitivity C-reactive protein (hsCRP) and tumor necrosis factor (TNF-α), markers of oxidative stress, such as malondialdehyde-modified LDL (MDA-LDL) and nitrotyrosine, molecules that relate to endothelial function, such as homocysteine and advanced glycation end products (AGE), and anthropometric measures, such as BMI, body weight and fat mass.

## 2 Case presentations

### 2.1 Therapeutic program

**Figure 1:**
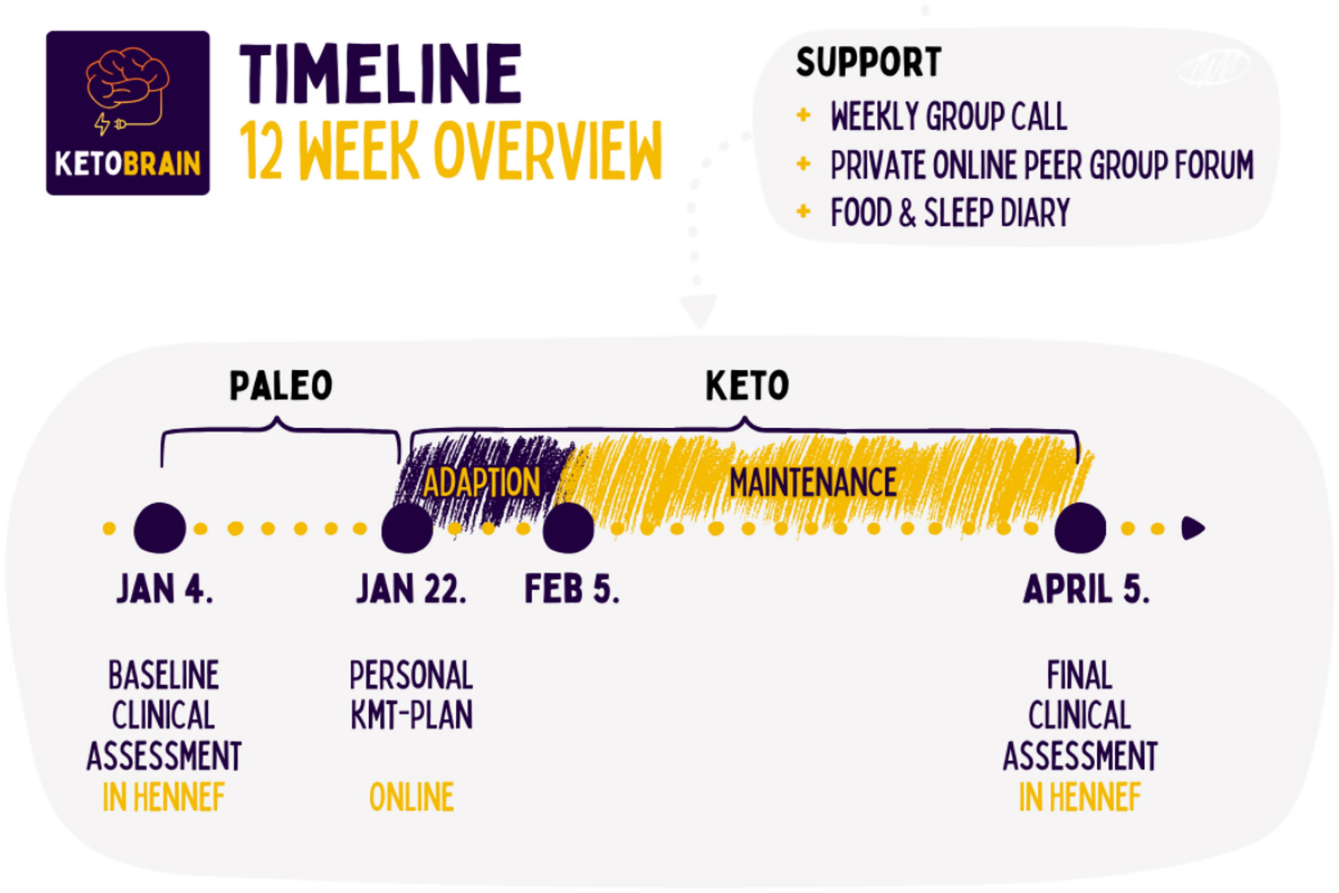
Graph showing the intervention timeline.

KetoBrain is a medically supervised program of the MOJO Institute for Regenerative Medicine, Hennef, Germany, focused on implementing ketogenic dietary therapy for people with psychiatric disorders. Between January 2024 and March 2024, 11 patients with bipolar disorder enrolled for the program and were placed on a personalized KD under the supervision of a medical doctor, a psychologist, and an intern ketogenic dietitian, under supervision of the treating medical doctor. In this therapeutic intervention (see Supplementary Data), KMT was used as a medical prescription, consisting of an individualized ketogenic diet, standardized micronutrient supplementation, and other lifestyle interventions such as physical exercise and community support provided by the program. Psychiatric medication and other therapeutic treatment remained unchanged throughout the 3-month duration of the program. Clinician support was provided weekly, mostly through digital group meetings (Figure 1).

Four participants withdrew from participation prior to program completion, two of which withdrew at the outset due to medical reasons. Two others stopped the intervention before completion because they did not respond well to the treatment. The program continued with seven remaining participants, whose initial and final clinical assessments are presented in this paper.

This case series received generous sponsorship from IMD Institut für Medizinische Diagnostik Berlin-Potsdam GbR, MOJO Institut für Regenerationsmedizin, Biocoach and Panifactum Lowcarb Food Gmbh, allowing all patients to participate in the program free of charge. Written informed consent was obtained from all participants to pursue KMT and to allow publications of de-identified data.

### 2.2 Case presentations

In this section, we present seven individual case descriptions with cardiometabolic outcomes, psychiatric outcomes and patient perspectives. For psychiatric measurements, German versions of Global Assessment of Functioning Scale (GAF) (49), Clinical Global Impression for Bipolar Disorder (CGI–BP) (50), Patient Health Questionnaire - 9 (PHQ–9) (51), Beck Depression Inventory II (BDI-II) (52), Hamilton-Depression Scale (HAM-D) (53) and Montgomery Asberg Depression Rating Scale (German version of MADRS) (54) were used. For more detailed patient profiles and psychiatric outcomes, we refer the reader to two forthcoming publications, which will discuss the effects of the intervention on psychiatric and microbiome health markers.

#### 2.2.1 Case 1

Case 1 is a 39-year-old single man with bipolar disorder II. He had a history of prediabetes, which was diagnosed in 2018, and presented with obesity (BMI 33.5 kg/m^2^). Since 2013 he had been treated with lithium (450 mg 1–0-2) and bupropion (300 mg 1–0-0). Since 2018 he has also taken metformin (1,000 mg 1–0-1). His laboratory assessment revealed a mixed dyslipidemic profile, with TG of 399 mg/dl, LDL-C of 146 mg/dl, apoB of 1.46 g/L, total cholesterol of 226 mg/dl, and HDL of 34 mg/dl. Being prediabetic, his HbA1c was elevated at 46 mmol/mol (6.4%). At the initiation of KMT 2:1, Case 1 intuitively preferred to adopt intermittent fasting and to consume only two meals per day (lunch and dinner). Describing himself as an emotional eater who frequently overeats, he was surprised to feel a remarkable decline in hunger and cravings. Within 1 week of keto-adaptation, he easily reached therapeutic nutritional ketosis with consistent serum BHB levels of between 1 and 4 mmol/L.

After the 3-month KD intervention, we found significant improvements in markers of obesity, prediabetes and inflammation, and mixed improvements in dyslipidemia. The patient lost 8 kg of body weight, BMI decreased to 30.9 kg/m^2^, body fat mass decreased from 39.0 kg to 34.3 kg and visceral fat percentage decreased from 10.6% to 8.8%. Moreover, HbA1c decreased to 5.1%, allowing the patient to discontinue metformin. The following dyslipidemic markers improved: TG decreased from 399 mg/dl to 202 mg/dl and HDL increased from 34 mg/dl to 42 mg/dl. However, total cholesterol increased to 272 mg/dl and LDL-C increased to 211 mg/dl. Despite this increase in LDL-C, apoB did not increase, staying quasi-constant from 1.46 g/L to 1.4 g/L. Accordingly, despite improvements in dyslipidemic markers, KMT did not reverse dyslipidemia, with LDL-C, TC and apoB still in the elevated range. Lp(a) slightly increased from 2.0 mg/dl to 3.1 mg/dl, but remained in a very low range. MDA-LDL, an important indicator of oxidative stress, decreased from 111 U/L to 70.4 U/L, now in the normal range. Inflammatory markers also improved with a significant reduction in hsCRP from 4.42 mg/L to 0.91 mg/L and a reduction in TNF-α from 10.6 to 9.5 pg/ml – albeit still in the elevated range. Nitrotyrosine remained in the standard range, despite an increase from <200 nmol/L to 572 nmol/L. Finally, laboratory assessments showed a concerning increase of homocysteine from 10.2 umol/L to 16 umol/L and a concerning increase of AGE from 36.1 ug/ml to 66.5 ug/ml.

The psychiatric outcomes of KMT were remarkable for Case 1. At the end of the intervention, he experienced profound improvements in emotional resilience, increased energy, better mental focus and stronger willpower. He went from a part-time job to working full-time. This was reflected in improvements in psychiatric assessments. His GAF, reflecting social, occupational and psychological functioning, improved from 60 to 100. CGI-BD-S, measuring symptom severity and treatment response in bipolar disorder, improved from 5 to 2 (1 = clinical remission, 2 = minimal symptoms). PHQ-9 and BDI-II, which are self-reported assessments measuring severity of depression, improved from 13 to 1 (PHQ-9) and from 20 to 3 (BDI-II), thereby achieving a “not depressed range.” Finally, HAM-D and MADRS, which are used by mental health professionals to measure severity of depression and treatment changes, also improved notably. HAM-D improved from 14 (mild depression) to 2 (clinically unremarkable) and MADRS improved from 22 (middle-grade depression) to 1, again ending up in the non-depressed range.

#### 2.2.2 Case 2

Case 2 is a 50-year-old married woman with rapid-cycling bipolar disorder II, which was diagnosed at age 30. Her medical history revealed sleep apnea, chronic pain syndrome, tinnitus and ADHD. She also had a history of irritable bowel syndrome, but she indicated that she was not troubled by this at present. Further complaints included itching, skin rashes, psoriasis, eczema, hives, acne and frequent headaches. She was treated with sertraline (50 mg 1–0-0) from 2023, lamotrigine (200 mg 1–0-1) from 2018 and quetiapine (50 mg 0–0–1-2) from 2023. At the initiation of KMT 1.5:1, Case 2 had difficulties reaching consistent serum BHB levels above 0.5 mmol/L. Upon shifting her KD ratio to 2:1, she still hardly reached 1 mmol/L. We surmise this may have been due to the impact of quetiapine, an antipsychotic that may lead to increased insulin secretion and plasma glucose levels, which both hinder ketogenesis. Throughout the 12-week-program, her blood BHB levels mostly fluctuated between 0.2 mmol/L and 1.0 mmol/L.

Metabolically, she was obese (BMI 34.7 kg/m^2^), with a body fat mass of 57.5 kg and visceral fat percentage of 11.2%. After 12 weeks of KMT, she had lost 14.5 kg of body weight, BMI decreased to 30.3 kg/m^2^, body fat mass decreased to 46.7 kg and visceral fat was reduced to 8.7%. Labs revealed a dyslipidemic profile, which improved significantly after the intervention. TG dropped from 245 mg/dl to 133 mg/dl, LDL-C decreased from 218 mg/dl to 203 mg/dl, apoB decreased from 1.86 g/L to 1.35 g/L, and total cholesterol decreased from 317 mg/dl to 270 mg/dl. However, Lp(a) was somewhat increased, going from 34.3 mg/dl to 38.7 mg/dl. In turn, MDA-LDL was reduced significantly, from 155 U/L to 72.1 U/L. Inflammatory markers improved slightly, with hsCRP going from 4.83 mg/L to 3.78 mg/L and TNF-α staying quasi-unchanged with an evolution from 9.8 pg/ml to 10 pg/ml – albeit still in the elevated range. Nitrotyrosine decreased from 326 nmol/L to <200 nmol/L, both being in the standard range. Finally, laboratory assessments again showed a concerning increase of homocysteine from 11 umol/L to 13.1 umol/L and a concerning increase of AGE from 25.7 ug/ml to 43.7 ug/ml.

At the end of the intervention, Case 2 also saw remarkable improvements in all psychiatric assessments. GAF improved from 50 to 80. CGI-BD-S improved from 5 to 2 (1 = clinical remission, 2 = minimal symptoms). PHQ-9 improved from 18 (indicating moderately severe depression) to 3 (indicating no depression). BDI-II improved from 42 (indicating severe depression) to 7 (indicating no depression). HAM-D improved from 21 (indicating moderately severe depression) to 5 (clinically unremarkable) and MADRS improved from 26 (indicating moderately severe depression) to 7 (indicating mild depression).

#### 2.2.3 Case 3

Case 3 is a 46-year-old married man with bipolar disorder II. Due to unwanted side-effects such as lethargy and fatigue, he had discontinued all medications under the supervision of his treating psychiatrist, 1 year prior to the present intervention. Six months after discontinuing his medications, and 4 months prior to the present intervention, he had a first 2.5 month experience with KMT. Since the shift to KMT, he had felt more energized, more emotionally balanced and resilient, and less irritable. During this first 2.5 month KMT, he had lost 11 kg. He decided to pursue KMT in the present intervention to optimize his KD and in the hope KMT would allow him to stay off medications. Until the time of this writing, he has been able to do so successfully.

Upon initiating the present KMT (1.5:1) intervention, Case 3 quickly reached therapeutic nutritional ketosis with consistent serum BHB levels between 1.0 and 3.0 mmol/L. Metabolically, he started the intervention with a BMI of 25.0 kg/m^2^, a fat mass of 29.3 kg and visceral fat percentage of 7.2%. Having already lost 11 kg in the previous 2.5-month KD phase in 2023, he lost less in this 3-month period, namely 3 kg. His BMI further decreased to 24.2 kg/m^2^, body fat mass further decreased to 26.8 kg, and visceral fat percentage decreased to 6.9%. While initial labs revealed a dyslipidemic profile, all elevated markers improved after the intervention. TG decreased from 60.3 mg/dl to 50.2 mg/dl, LDL-C decreased from 178 mg/dl to 145 mg/dl, apoB decreased from 1.42 g/L to 1.03 g/L and total cholesterol decreased from 215 mg/dl to 183 mg/dl. HDL remained unchanged at 35 mg/dl. Of note was also a decrease in Lp(a), from 10.4 mg/dl to 8.1 mg/dl, and of MDA-LDL, from 77.4 U/L to 38.7 U/L. In turn, inflammatory markers also improved, with hsCRP decreasing from 2.27 mg/L to 1.01 mg/L and TNF-α decreasing from 7.7 pg/ml to 6.2 pg/ml. Baseline nitrotyrosine was strongly elevated at 3043 nmol/L and greatly decreased to 1463 nmol/L, albeit still in the elevated range. Finally, we found a decrease in homocysteine from 17 umol/L to 14 umol/L, albeit both in the elevated range, and a concerning increase of AGE from 28.7 ug/ml to 45.7 ug/ml, albeit both in the standard range.

After the intervention, Case 3 showed improvements across all psychiatric assessments. His GAF improved from 95 to 100. CGI-BD-S improved from 4 to 2. PHQ-9 and BDI-II improved from 6 to 2 (PHQ-9) and from 6 to 2 (BDI-II). HAM-D improved from 5 to 3 and MADRS improved from 7 to 1. Case 3 continues to follow KMT to this day, considering it a life-changing intervention, his friends and family attesting to its favorable therapeutic effects.

#### 2.2.4 Case 4

Case 4 is a 47-year-old woman with bipolar disorder II, which was diagnosed in 2023, at 46 years of age. She has been treated with lamotrogine (50 mg 1–0-1) since her diagnosis in 2023 and escitalopram (15 mg) since having had long covid in February 2022. At the initiation of KMT 1.5:1, Case 4 quickly reached consistent serum BHB levels of 1.0 mmol/L. Throughout the 12-week-program, her blood BHB levels mostly fluctuated between 0.5 and 1.0 mmol/L.

Metabolically, she began the intervention with a BMI of 22.8 kg/m^2^, a body fat mass of 18.6 kg and visceral fat percentage of 4.5%. After the intervention, her weight decreased to 54.5 kg, BMI decreased to 21.8 kg/m^2^, her fat mass decreased to 17.5 kg, and her visceral fat percentage decreased to 4.1%. Labs revealed a dyslipidemic profile with elevated LDL-C of 123 mg/dl and total cholesterol of 240 mg/dl. After the intervention, both markers further increased to 141 mg/dl (LDL-C) and 240 mg/dl (total cholesterol). However, her baseline apoB of 0.92 g/L remained quasi unchanged at 0.91 g/L, again marking discordance between LDL-C and apoB. HDL increased from 105 mg/dl to 113 mg/dl, and triglycerides slightly increased from 63.1 mg/dl to 66.6 mg/dl. Also, Lp(a) decreased from an elevated 92.8 mg/dl to 56.8 mg/dl, albeit still in the elevated range. MDA-LDL and nitrotyrosine, both markers of oxidative stress, decreased significantly from 73.3 U/L to 42.8 U/L (MDA-LDL) and from 926 nmol/L to 432 nmol/L (nitrotyrosine). Inflammatory markers also improved after the intervention: baseline hsCRP of <0.3 mg/L remained unchanged, while TNF-α decreased from 5.6 pg/ml to 4.3 pg/ml. Finally, both homocysteine and AGE again showed concerning changes with homocysteine increasing from 6.1 umol/L to 12.4 umol/L and AGE increasing from 28.5 ug/ml to 64.1 ug/ml.

After the intervention, Case 4 showed improvements across all psychiatric assessments. Her GAF improved from 60 to 100. CGI-BD-S improved from 3 to 2. PHQ-9 and BDI-II improved from 9 to 0 (PHQ-9) and from 13 to 1 (BDI-II). HAM-D improved from 9 to 0 and MADRS improved from 8 to 0.

#### 2.2.5 Case 5

Case 5 is a 34-year-old married woman with bipolar disorder I, which was diagnosed at 32 years of age, and comorbid anxiety disorder, which was diagnosed at 19 years of age. Since 2022, she has been treated with lithium (450 mg 1–0-1.5) and quetiapine (150 mg 0–0-1). Metabolically, she had a BMI of 26.6 kg/m^2^, with a body fat mass of 30 kg and visceral fat percentage of 4.1%. After the intervention, she lost 2.9 kg, her BMI decreased to 25.7 kg/m^2^, body fat mass decreased to 27.9 kg and visceral fat decreased to 3.5%. She too presented with dyslipidemia at the outset, but her elevated markers evolved heterogeneously. While total cholesterol increased from 219 mg/dl to 234 mg/dl, HDL slightly decreased from 64 mg/dl to 63 mg/dl, and LDL-C increased from 151 mg/dl to 179 mg/dl, her apoB remained unchanged at 1.01 g/L, again marking discordance between LDL-C and apoB. Favorable changes included a decrease in Lp(a) from 7.3 mg/dl to 4.9 mg/dl, and a decrease in TG from 51.1 mg/dl to 33.8 mg/dl. Both markers of oxidative stress also improved with MDA-LDL decreasing from 67.2 U/L to 54.9 U/L and nitrotyrosine decreasing from 438 nmol/L to 315 nmol/L. Inflammatory markers also improved with hsCRP remaining unchanged at <0.3 mg/L and TNF-α decreasing from 13.1 pg/ml to 8.9 pg/ml. Finally, both homocysteine and AGE again increased unfavorably with homocysteine rising from 6.6 umol/L to 11.3 umol/L and AGE increasing from 18.6 ug/ml to 53.5 ug/ml.

After the intervention, Case 5 also showed improvements across all psychiatric assessments. Her GAF improved from 60 to 100. CGI-BD-S improved from 4 to 1. PHQ-9 and BDI-II improved from 3 to 2 (PHQ-9) and from 8 to 3 (BDI-II). HAM-D improved from 7 to 0 and MADRS improved from 9 (mild depression) to 1 (no depression).

#### 2.2.6 Case 6

Case 6 is a 29-year-old man with bipolar disorder I, which was diagnosed in 2015, at 20 years of age. Medical history was notable for a severe case of Lyme disease immediately preceding the onset of his first depressive episode at the age of 18, with the formal diagnosis of bipolar disorder following 3 years later. He was treated with valproate (1,000 mg 1–0-1) and sertraline (200 mg 1–0-0). At the initiation of KMT 1.5:1, Case 6 quickly reached consistent serum BHB levels between 1.0–2.0 mmol/L. Throughout the 12-week-program, his blood BHB levels mostly fluctuated between 1.0 and 3.0 mmol/L.

Metabolically, he had a BMI of 26.5 kg/m^2^, a body fat mass of 25.2 kg and visceral fat percentage of 4%. After 12 weeks of KMT, he had lost 1.9 kg of bodyweight, BMI decreased to 25.9 kg/m^2^, body fat mass decreased to 19.2 kg and visceral fat decreased to 3.5%. Labs revealed a dyslipidemic profile, with elevated levels of total cholesterol (237 mg/dl) and LDL-C (178 mg/dl). After the intervention, both dyslipidemic markers increased, with total cholesterol reaching 242 mg/dl and LDL-C reaching 192 mg/dl. However, apoB decreased from 1.33 g/L to 1.17 g/L, again showing remarkable discordance between LDL-C and apoB. Other favorable lipid changes include an increase of HDL from 52 mg/dl to 54 mg/dl, a lowering of TG from 70 mg/dl to 59.1 mg/dl and a decrease in Lp(a) from 24.3 mg/dl to 16 mg/dl. MDA-LDL also improved, decreasing from 108 U/L to 79 U/L. On the other hand, nitrotyrosine increased from 425 nmol/L to 799 nmol/L, now in the elevated range. Inflammatory markers evolved positively with hsCRP lowering from 1.2 mg/L to 0.3 mg/L and TNF-α slightly decreasing from 8.7 pg/ml to 8.4 pg/ml, albeit still in the elevated range. Baseline homocysteine of 14.7 umol/L remained elevated at 14.7 umol/L, and baseline AGE again increased unfavorably from 27.6 ug/ml to 56.3 ug/ml.

After the intervention, Case 6 saw mixed changes in psychiatric assessments. While some assessments improved, others remained unchanged and some slightly worsened. For this reason, we do not consider Case 6 an unequivocal respondent to this intervention. While GAF improved from 80 to 100, CGI-BD-S slightly worsened from 3 to 4. PHQ-9 slightly worsened from 1 to 2, but this is still in the “healthy” range. BDI-II improved from 9 (minimal depression) to 5 (no depression). HAM-D slightly worsened from 2 to 3, both being in the “clinically unremarkable” range, while MADRS slightly improved from 3 to 2, both indicating “no depression.”

#### 2.2.7 Case 7

Case 7 is a 50-year-old married man with bipolar disorder I, which was diagnosed in 2004, at 30 years of age. His medical history was notable for ankylosing spondylitis (Morbus Bechterew) and recurrent migraines. He was treated with quetiapine (275 mg 0–0-1) and valproate (200 mg 0–0-1). At the beginning of our KMT intervention, Case 7 chose to adopt a carnivore KD, with virtually no carbohydrate intake. We agreed to this, on the condition his serum BHB levels would consistently reach between 1.0 mmol/L and 5.0 mmol/L. Throughout the intervention, this was consistently the case.

Metabolically, he had a BMI of 22.3 kg/m^2^, a body fat mass of 16.2 kg and visceral fat percentage of 5.6%. After 12 weeks of KMT, he had gained 4.4 kg (3.8 kg of which was muscle mass), BMI increased to 23.6 kg/m^2^, body fat mass increased to 17.5 kg and visceral fat percentage also increased to 6.9%. Labs revealed a dyslipidemic profile, with baseline elevated markers including a total cholesterol of 224 mg/dl and LDL-C of 162 mg/dl. At the end of the intervention, total cholesterol increased to 258 mg/dl, and LDL-C increased to 185 mg/dl. Further concerning changes included an increase of baseline TG from 51.1 mg/dl to 111 mg/dl and an increase in baseline apoB from 1.18 g/L to 1.35 g/L. Conversely, baseline HDL increased from 54 mg/dl to 61 mg/dl and Lp(a) decreased from 46.5 mg/dl to 44 mg/dl. MDA-LDL also decreased from 84.3 U/L to 52.4 U/L, while baseline nitrotyrosine of <200 nmol/L remained unchanged at <200 nmol/L. Effects on inflammatory markers were also mixed, with hsCRP slightly increasing from 1.14 mg/L to 1.3 mg/L and TNF-α slightly decreasing from 70.9 pg/ml to 69.9 pg/ml, being still markedly elevated. Finally, homocysteine decreased from 10.9 umol/L to 8.6 umol/L and AGE increased from 38.6 ug/ml to 89.9 ug/ml.

Psychiatric assessments indicated that Case 7 was a non-respondent to KMT. While some assessments either improved minimally and some assessments remained unchanged, others worsened. Initial GAF score of 60 improved to 70, but initial CGI-BD-S score remained unchanged (4). The PHQ score slightly worsened from 8 to 9, but this remained within the “clinically unremarkable” range. BDI-II slightly improved from 8 to 7, both indicating “no depression.” HAM-D remained unchanged, both initial and final assessments scoring 10, indicating “mild depression.” Finally, the MADRS assessment gave a worse result at the end of the intervention, going from 6 (“no depression”) to 12 (“mild depression”).

## 3 Discussion

### 3.1 Main findings

In this section, we present mean intervention outcomes using box plots, which show the evolution of all patients. All cardiometabolic outcomes are displayed in Table 1. All data and code are available upon request.

**Table 1:** Changes in laboratory findings.

Outcome	n	Pre-KD, Mean	Pre-KD, SD	Post-KD, mean	Post-KD, SD	Change	Percent change
**AGE, ug/ml**	7	29.11	6.63	59.97	15.69	30.86	105.99
**Homocysteine, umol/L**	7	10.93	3.95	12.87	2.43	1.94	17.78
**LDL-C, mg/dl**	7	165.14	30.21	179.43	27.09	14.29	8.65
**HDL-C, mg/dl**	7	56.57	23.89	59.57	25.57	3.00	5.30
**Cholesterol, mg/dl**	7	239.71	35.25	246.86	31.75	7.14	2.98
**LDL/HDL ratio**	7	3.37	1.31	3.41	1.19	0.04	1.27
**Weight, kg**	7	88.34	19.78	84.29	16.10	−4.05	−4.58
**TNF-a, pg/ml**	7	18.06	23.42	16.74	23.53	−1.31	−7.28
**Visceral fat (%)**	7	6.74	3.05	6.06	2.34	−0.69	−10.17
**Apo-lipoprotein B, g/L**	7	1.31	0.32	1.17	0.20	−0.14	−10.46
**Fat mass, kg**	7	30.83	14.00	27.13	10.67	−3.70	−12.00
**Lipoprotein (a), mg/dl**	7	31.09	31.47	24.51	21.64	−6.57	−21.14
**Nitrotyrosine, nmol/L**	7	794.00	1021.68	568.71	448.66	−225.29	−28.37
**Triglycerides, mg/dl**	7	134.23	135.92	93.67	59.14	−40.56	−30.21
**MDA-LDL, U/L**	7	96.66	30.70	59.89	16.93	−36.77	−38.04
**HsCRP, mg/L**	7	2.07	1.87	1.13	1.24	−0.94	−45.37

#### 3.1.1 Mean effects on lipid metabolism

**Figure 2:**
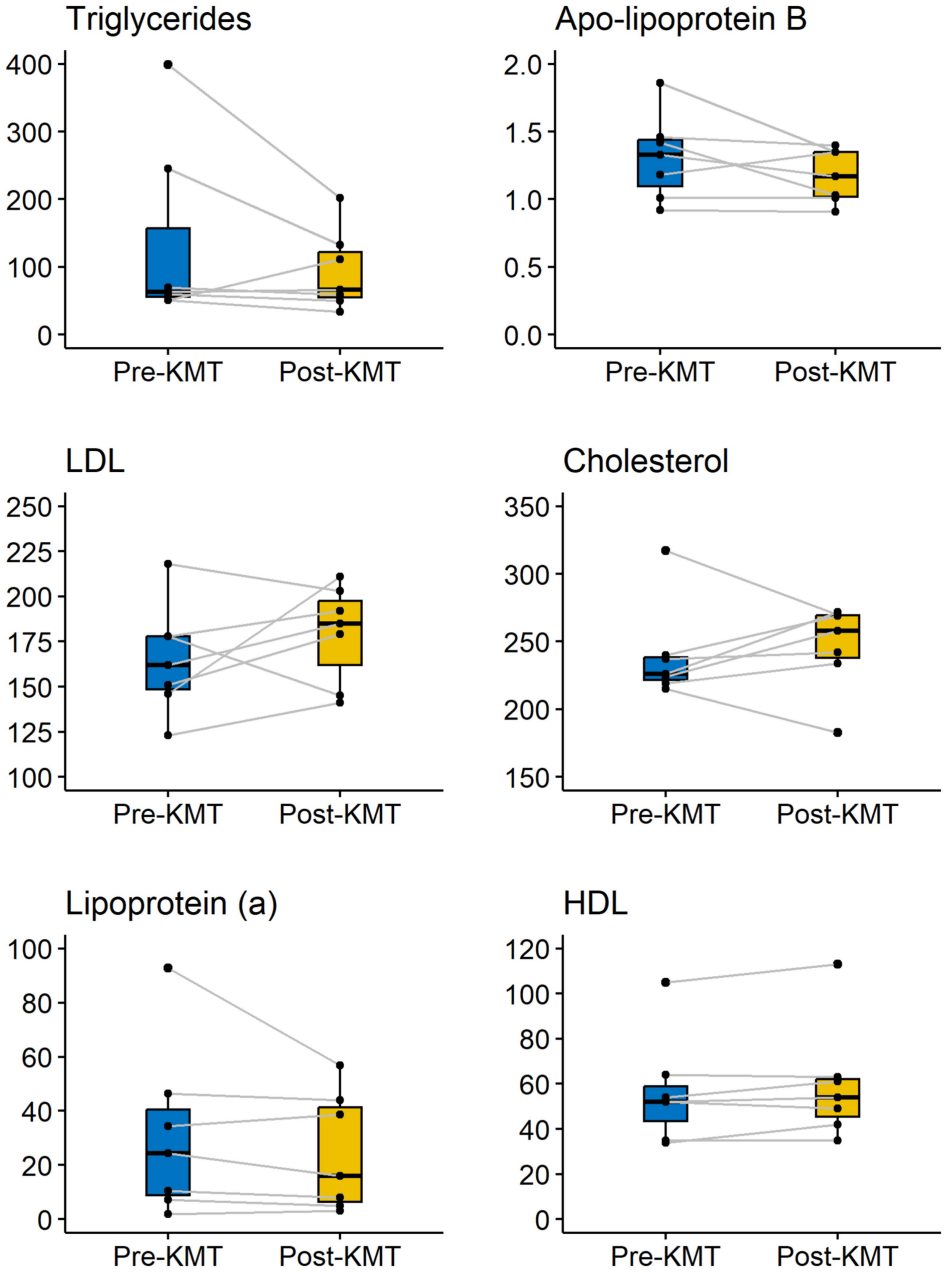
Changes in lipid metabolism over time.

Following the KD intervention, mean (SD) effects on lipid metabolism showed largely favorable changes. While most of these were predictable, our findings also showed an unexpected discordance between LDL-C and apoB (Figure 2).

First of all, in line with the literature to date, one would expect KMT to have the following positive effects on lipid metabolism: a reduction in TG, an increase in HDL-C, and possibly a reduction in Lp(a) (47, 55, 56). Our mean results were in line with these expectations. Mean TG was lowered by 40.56 mg/dl, which is a 30% reduction over the span of 3 months. Mean HDL-C was slightly increased by 3 mg/dl, which is a 5% increase. Of note, mean Lp(a) was lowered by 6.57 mg/dl, which is a 21% reduction.

Second, based on the current literature, we know KMT may have a variable effect on LDL-C and total cholesterol, potentially improving or worsening hyperlipidemia (47, 48, 55). If an animal-based KD is adopted, one may reasonably expect an increase in both markers. This was also the case in our findings, with both LDL-C and TC increased in 5 out of 7 patients. We found a mean increase of LDL-C of 14.29 mg/dl, which is a 8.6% increase, and a mean increase of total cholesterol of 7.14 mg/dl, which is a 3% increase. It should be noted that all participants adopted a KD high in saturated fat, against our dietary recommendation to consume a Mediterranean ketogenic diet, consisting of 50% MUFA, 25% PUFA and 25% SFA.

Third, based on the current literature, one would expect to find a high correlation between changes in LDL-C and apoB. As the 2019 European Atherosclerosis Society/European Society of Cardiology Guidelines state, it is generally estimated that only in 20% of patients there may be discordance between measured LDL-C and apoB levels (57). This high correlation between LDL-C and apoB is one of the reasons why apoB is often not measured in routine clinical care. When recommended by guidelines and consensus groups, measurement of apoB has been prioritized or restricted to patients with hypertriglyceridaemia, the presumption being cholesterol-depleted apoB particles are only common in such individuals.

In contrast to the high correlation between apoB and LDL-C, our case series highlights a remarkable discordance between changes in LDL-C and apoB. In fact, while mean LDL-C increased by 14 mg/dl (a 8.6% increase), mean apoB was decreased by 14 mg/dl (0.14 g/L, as indicated in the table above), which is a reduction of 10.46%. Indeed, whereas 5 out of 7 patients saw their LDL-C increase, only 1 patient saw his apoB increase, 2 patients saw their apoB decrease, and 4 saw their apoB unchanged. Of the 5 patients who had increased LDL-C, 3 saw their apoB remain unchanged, 1 saw his apoB decrease and only 1 saw his apoB increase.

The observed increase in LDL-C without a change in apoB, or with a decrease in apoB, may indicate a shift in the composition of LDL particles, from small-dense LDL (sdLDL) particles towards large, buoyant (lbLDL) LDL particles. Given such a shift, LDL particles would contain more cholesterol per particle (hence the increase in LDL-C) but the total number of LDL particles (and hence apoB) would not have increased, or even have decreased. This suggests that in at least 4 patients, our intervention led to a shift in LDL particle size, which would increase LDL concentration but not apoB count. Whether or not such a shift reduces overall cardiovascular risk, has been a heated topic of debate (58–65). As it stands, the markers of apoB and LDL particle number are considered stronger predictors of cardiovascular risk than LDL-C. Thus, the observed mean increase in LDL-C should be seen in light of the observed mean decrease in apoB. Potential discordance between LDL-C and apoB underlines the importance of measuring both markers to accurately evaluate individual cardiometabolic effects of KMT.

Finally, it is noteworthy that triglycerides did not add to the predictive value of changes in apoB. Contrary to current expectations, discordance between LDL-C and apoB did not correlate with elevated TG. Of the 4 patients who saw discordance between both markers, only one had elevated TG, whereas all others had TG below 66.6 mg/dl.

#### 3.1.2 Mean effects on markers of inflammation

**Figure 3:**
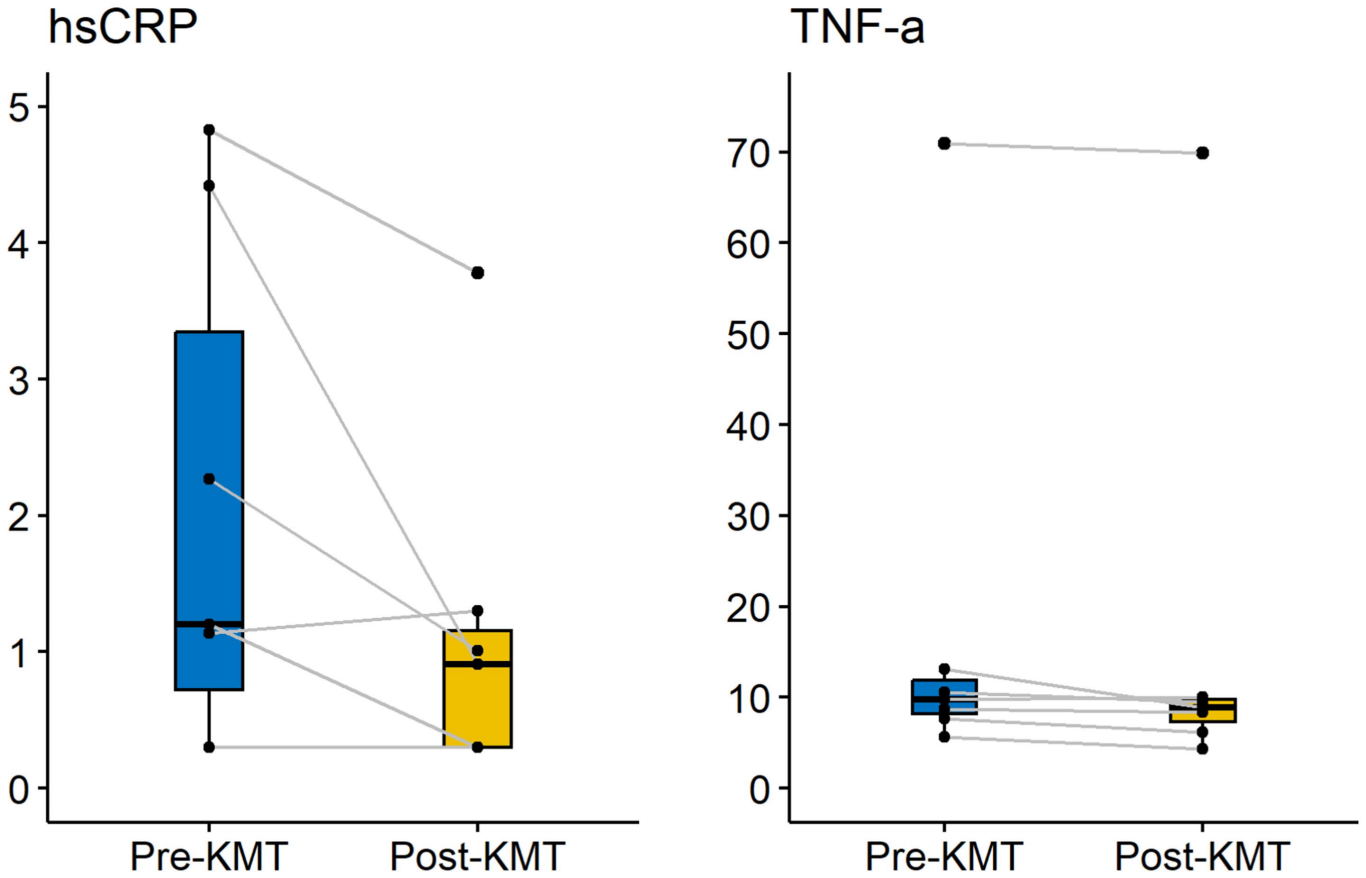
Changes in inflammatory markers over time.

Since mitochondria and metabolism are fundamental regulators of immune function, it should come as no surprise that metabolic interventions can have a profound impact on immunity and inflammation (66). KDs in particular have consistently been shown to lower markers of inflammation such as C-reactive protein (CRP), tumor necrosis factor-alpha (TNF-α) and interleukin (IL)-6 (67). After our intervention, mean hsCRP was reduced by 0.94 mg/L, which is a reduction of 45%, and mean TNF-α was reduced by 1.31 pg/ml, which is a reduction of 7% (Figure 3).

#### 3.1.3 Mean effects on markers of oxidative stress

**Figure 4:**
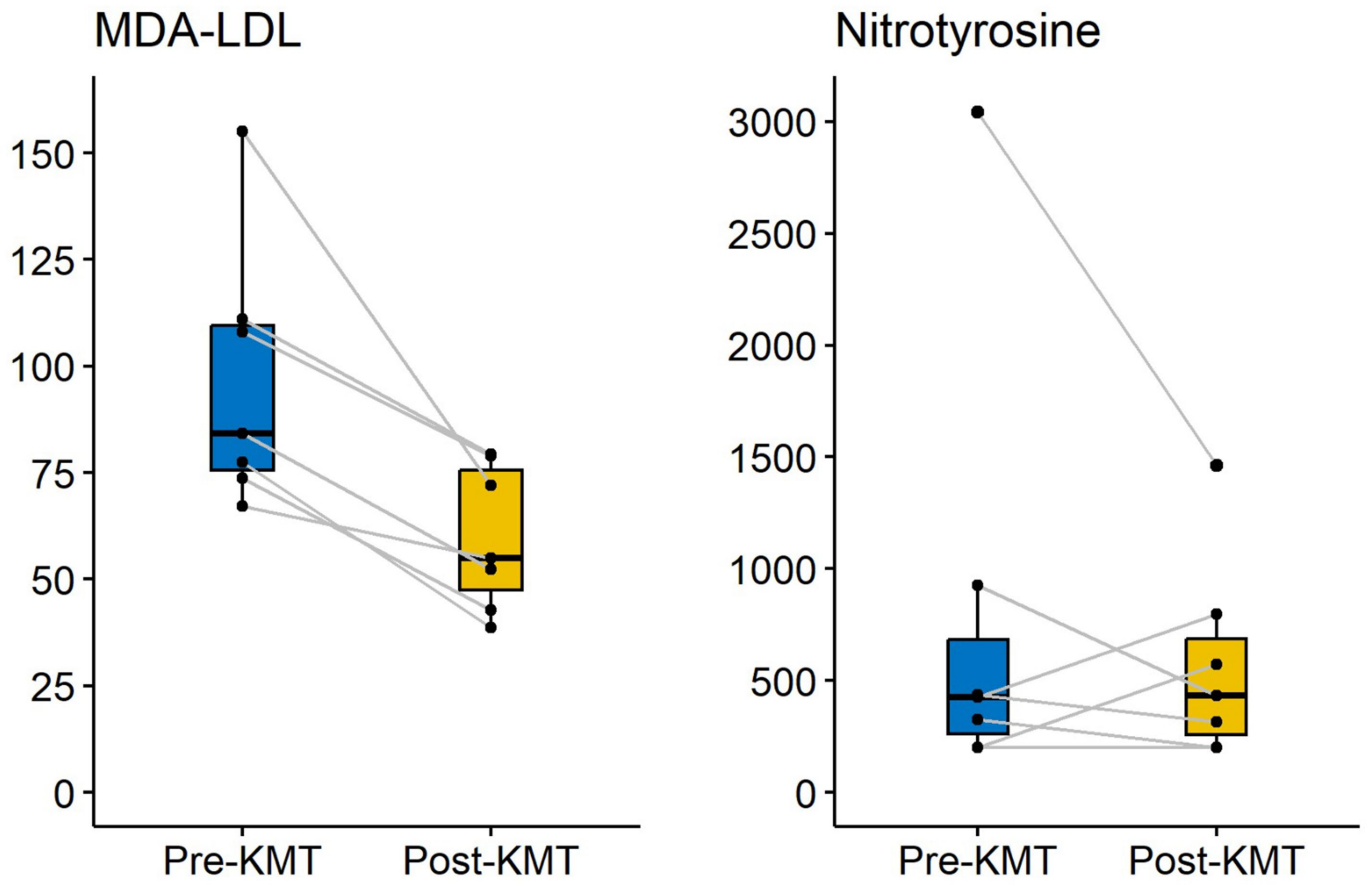
Changes in markers of oxidative stress over time.

Chronic oxidative stress is found in most chronic diseases, ranging from heart disease and type 2 diabetes to schizophrenia and bipolar disorder (68). Our case series looked at two key biomarkers for systemic oxidative stress, namely malondialdehyde-modified LDL (MDA-LDL) and nitrotyrosine. Malondialdehyde is a result of oxidative fatty acid degeneration. It has cytotoxic effects and attacks LDL particles, with MDA-LDL as a result. While LDL-C is an important risk factor for atherosclerosis, its oxidized forms, such as MDA-LDL, are especially atherogenic. Some studies have even suggested MDA-LDL is a superior marker of ASCVD compared to other commonly used lipid markers (69). We found a mean reduction of MDA-LDL of 36.77 U/L, which is decrease of 38% (Figure 4).

Persistent oxidative stress can lead to excessive formation of reactive nitrogen species (RNS), also referred to as nitrosative stress (NSS). RNS can lead to cell damage through the generation of nitrotyrosine, causing toxic effects on cells, much like oxidative stress. Nitrosative stress is a highly oxidizing, pathological condition that is found to participate in many cardiometabolic diseases such as atherosclerosis, diabetes, hypertension and endothelial dysfunction (70). After our KMT intervention, we have found a mean reduction in serum nitrotyrosine levels of 225 nmoL/L, a reduction of 28%.

#### 3.1.4 Mean effects on homocysteine and advanced glycation end products

As noted in the case presentations above, laboratory assessments showed concerning increases in homocysteine and advanced glycation end products (AGE) throughout our cases. Given the impact of both markers on cardiovascular risk, these findings are remarkable. Mean baseline homocysteine was already elevated, with 10.9 umol/L. After the intervention, mean homocysteine increased to 12.9 umol/L, an 18% increase. This phenomenon may be attributed to an increased dietary intake of methionine without a corresponding and sufficient supply of essential cofactors such as vitamin B6, vitamin B9, vitamin B2, choline, zinc and/or magnesium, which are required for proper function of the methylation cycle. These co-factors can be lacking in a non-supplemented ketogenic diet without organ meats. Mean baseline AGE was in the standard range, with 29 ug/ml. However, after the intervention mean AGE was increased to 60 ug/ml, a 106% increase. This may have been caused by maillard reactions of fried, roasted and grilled meats, a depleted glyoxalase system caused by a relative deficiency of vitamin B1 (often lacking in organ-free ketogenic diets), general anti-oxidant deficiency, or physiological insulin resistance often seen in ketogenic diets, potentially leading to higher blood glucose and AGE formation.

#### 3.1.5 Mean effects on body weight, visceral fat and fat mass

Mean weight reduction was 4 kg (−4.6%), mean visceral fat reduction was 0.69% (−10%) and mean fat mass reduction was 3.7 kg (−12%).

### 3.2 Limitations

Although this case series confirms several potential therapeutic effects of KMT on a range of cardiometabolic health markers, it is important to note the following limitations of this retrospective study. First of all, a case series such as this is an observational, descriptive study that allows for no generalizations to be made. The discussed intervention did not utilize a control group and its cohort is far too small to allow any meaningful statistical inference to be made. Thus, this study does not intend to provide evidence for causality. Instead, it intends to share remarkable experiences with other clinicians and to generate hypotheses for future research. Furthermore, it is important to note that beyond the ketogenic diet, this intervention also utilised micronutrient supplementation and intensive peer group support, both of which may also have contributed to improving patient outcomes.

## 4 Conclusion

In conclusion, this case series highlights remarkable improvements in a broad range of cardiometabolic health markers, including lipid metabolism, inflammation, oxidative stress and anthropometric measures. Regarding lipid metabolism, we found a reduction in mean TG by 40.6 mg/dl (−30%), a remarkable reduction in mean Lp(a) by 6.6 mg/dl (−21%), a reduction in mean apoB by 0.14 g/L (−10%) and an increase in mean HDL-C by 3 mg/dl (+5%). Regarding inflammation, we found a reduction of mean hsCRP of 0.94 mg/L (−45%) and a reduction in mean TNF-α by 1.31 pg/ml (−7%). Regarding oxidative stress, we found a reduction in mean MDA-LDL of 36.77 U/L (−38%) and a reduction in mean nitrotyrosine of 225 nmol/L (−28%). Finally, regarding anthropometric measures, we found a mean weight reduction of 4 kg (−4.6%), a mean visceral fat reduction of 0.69% (−10%) and a mean fat mass reduction of 3.7 kg (−12%).

However, some concerning effects were also observed. Of note, mean homocysteine levels increased by 1.94 umol/L (+18%) and mean AGE levels increased by 30.9 ug/ml (+106%). Moreover, mean LDL-C was increased by 14 mg/dl (+9%) and mean total cholesterol was increased by 7 mg/dl (+3%). Given recent consensus that apoB more accurately reflects the atherogenic burden of lipoproteins when compared to LDL-C, this mean increase in LDL-C should be seen in the context of the observed mean decrease in apoB. Potential discordance between LDL-C and apoB underlines the importance of measuring both markers to accurately evaluate individual cardiometabolic effects of KMT.

Based on these findings, we conclude that comprehensive Ketogenic Metabolic Therapy provided to outpatients with bipolar disorder can be beneficial in improving a broad range of cardiometabolic health markers, including lipid metabolism, inflammation, oxidative stress and anthropometric measures. Tentatively, these findings suggest that at least a proportion of patients with bipolar disorder may find remarkable improvements in cardiometabolic health adopting a metabolic treatment such as the ketogenic diet. However, potentially concerning effects on markers such as homocysteine and AGE call for well-formulated, individualized KDs. Further research is needed to explore these effects and to investigate optimal variants of KMT that may best address these concerns.

## Data Availability

The original contributions presented in the study are included in the article/Supplementary material, further inquiries can be directed to the corresponding author.

## References

[1] Osby U Brandt L Correia N Ekbom A Sparén P. Excess mortality in bipolar and unipolar disorder in Sweden. Arch Gen Psychiatry. (2001) 58:844–50. doi: 10.1001/archpsyc.58.9.844, 11545667

[2] Cassidy F Ahearn E Carroll BJ. Elevated frequency of diabetes mellitus in hospitalized manic-depressive patients. Am J Psychiatry. (1999) 156:1417–20. doi: 10.1176/ajp.156.9.1417, 10484954

[3] Lilliker SL. Prevalence of diabetes in a manic-depressive population. Compr Psychiatry. (1980) 21:270–5. doi: 10.1016/0010-440X(80)90030-9, 7398250

[4] McIntyre RS Konarski JZ Misener VL Kennedy SH. Bipolar disorder and diabetes mellitus: epidemiology, etiology, and treatment implications. Ann Clin Psychiatry. (2005) 17:83–93. doi: 10.1080/10401230590932380, 16075661

[5] Plana-Ripoll O Pedersen CB Agerbo E Holtz Y Erlangsen A Canudas-Romo V . A comprehensive analysis of mortality-related health metrics associated with mental disorders: a nationwide, register-based cohort study. Lancet. (2019) 394:1827–35. doi: 10.1016/S0140-6736(19)32316-5, 31668728

[6] Crump C Sundquist K Winkleby MA Sundquist J. Comorbidities and mortality in bipolar disorder: a Swedish national cohort study. JAMA Psychiatr. (2013) 70:931–9. doi: 10.1001/jamapsychiatry.2013.1394, 23863861

[7] Liu NH Daumit GL Dua T Aquila R Charlson F Cuijpers P . Excess mortality in persons with severe mental disorders: a multilevel intervention framework and priorities for clinical practice, policy and research agendas. World Psychiatry. (2017) 16:30–40. doi: 10.1002/wps.20384, 28127922 PMC5269481

[8] Weiner M Warren L Fiedorowicz JG. Cardiovascular morbidity and mortality in bipolar disorder. Ann Clin Psychiatry. (2011) 23:40–7. 21318195 PMC3190964

[9] Westman J Hällgren J Wahlbeck K Erlinge D Alfredsson L Ösby U. Cardiovascular mortality in bipolar disorder: a population-based cohort study in Sweden. BMJ Open. (2013) 3:e002373. doi: 10.1136/bmjopen-2012-002373, 23604348 PMC3641504

[10] Vendsborg PB Bech P Rafaelsen OJ. Lithium treatment and weight gain. Acta Psychiatr Scand. (1976) 53:139–47. doi: 10.1111/j.1600-0447.1976.tb00067.x, 1251759

[11] Sachs G Bowden C Calabrese JR Ketter T Thompson T White R . Effects of lamotrigine and lithium on body weight during maintenance treatment of bipolar I disorder. Bipolar Disord. (2006) 8:175–81. doi: 10.1111/j.1399-5618.2006.00308.x, 16542188

[12] Hermida OG Fontela T Ghiglione M Uttenthal LO. Effect of lithium on plasma glucose, insulin and glucagon in normal and streptozotocin-diabetic rats: role of glucagon in the hyperglycaemic response. Br J Pharmacol. (1994) 111:861–5. doi: 10.1111/j.1476-5381.1994.tb14817.x, 8019763 PMC1910068

[13] Huang TL Chen JF. Serum lipid profiles and schizophrenia: effects of conventional or atypical antipsychotic drugs in Taiwan. Schizophr Res. (2005) 80:55–9. doi: 10.1016/j.schres.2005.05.001, 15964176

[14] Henderson DC Cagliero E Gray C Nasrallah RA Hayden DL Schoenfeld DA . Clozapine, diabetes mellitus, weight gain, and lipid abnormalities: a five-year naturalistic study. Am J Psychiatry. (2000) 157:975–81. doi: 10.1176/appi.ajp.157.6.975, 10831479

[15] Spivak B Lamschtein C Talmon Y Guy N Mester R Feinberg I . The impact of clozapine treatment on serum lipids in chronic schizophrenic patients. Clin Neuropharmacol. (1999) 22:98–101. doi: 10.1097/00002826-199903000-00006, 10202605

[16] Gaulin BD Markowitz JS Caley CF Nesbitt LA Dufresne RL. Clozapine-associated elevation in serum triglycerides. Am J Psychiatry. (1999) 156:1270–2. doi: 10.1176/ajp.156.8.1270, 10450273

[17] Osser DN Najarian DM Dufresne RL. Olanzapine increases weight and serum triglyceride levels. J Clin Psychiatry. (1999) 60:767–70. doi: 10.4088/JCP.v60n1109, 10584766

[18] Guo JJ Keck PE Jr Corey-Lisle PK Li H Jiang D Jang R . Risk of diabetes mellitus associated with atypical antipsychotic use among patients with bipolar disorder: A retrospective, population-based, case-control study. J Clin Psychiatry. (2006) 67:1055–61. doi: 10.4088/JCP.v67n0707, 16889448

[19] Lambert BL Chou CH Chang KY Tafesse E Carson W. Antipsychotic exposure and type 2 diabetes among patients with schizophrenia: a matched case-control study of California Medicaid claims. Pharmacoepidemiol Drug Saf. (2005) 14:417–25. doi: 10.1002/pds.1092 15786516

[20] Ollendorf DA Joyce AT Rucker M. Rate of new-onset diabetes among patients treated with atypical or conventional antipsychotic medications for schizophrenia. Medscape Gen Med. (2004) 6:5. PMC1140712 15208518

[21] Sernyak MJ Gulanski B Rosenheck R. Undiagnosed hyperglycemia in patients treated with atypical antipsychotics. J Clin Psychiatry. (2005) 66:1463–7. doi: 10.4088/JCP.v66n1117, 16420085

[22] Carlson C Hornbuckle K Delisle F Kryzhanovskaya L Breier A Cavazzoni P. Diabetes mellitus and antipsychotic treatment in the United Kingdom. Eur Neuropsychopharmacol. (2005) 16:366–75. doi: 10.1016/j.euroneuro.2005.11.002 16356695

[23] Gianfrancesco F White R Wang RH Nasrallah HA. Antipsychotic-induced type 2 diabetes: evidence from a large health plan database. J Clin Psychopharmacol. (2003) 23:328–35. doi: 10.1097/01.jcp.0000085404.08426.3a, 12920407

[24] Calkin C van de Velde C Ruzickova M. Can body mass index help predict outcome in patients with bipolar disorder? Bipolar Disord. (2009) 11:650–6. doi: 10.1111/j.1399-5618.2009.00730.x, 19689507 PMC3544930

[25] Kemp DE Gao K Chan PK Ganocy SJ Findling RL Calabrese JR. Medical comorbidity in bipolar disorder: relationship between illnesses of the endocrine/metabolic system and treatment outcome. Bipolar Disord. (2010) 12:404–13. doi: 10.1111/j.1399-5618.2010.00823.x, 20636638 PMC2913710

[26] Thompson WK Kupfer DJ Fagiolini A Scott JA Frank E. Prevalence and clinical correlates of medical comorbidities in patients with bipolar I disorder: analysis of acute-phase data from a randomized controlled trial. J Clin Psychiatry. (2006) 67:783–8. doi: 10.4088/jcp.v67n0512 16841628

[27] Yim CY Soczynska JK Kennedy SH Woldeyohannes HO Brietzke E McIntyre RS. The effect of overweight/obesity on cognitive function in euthymic individuals with bipolar disorder. Eur Psychiatry. (2012) 27:223–8. doi: 10.1016/j.eurpsy.2011.02.004, 21570263

[28] Kuswanto CN Sum MY Yang GL Nowinski WL McIntyre RS Sim K. Increased body mass index makes an impact on brain white-matter integrity in adults with remitted first-episode mania. Psychol Med. (2014) 44:533–41. doi: 10.1017/S0033291713000858, 23731622

[29] Calkin CV Ruzickova M Uher R Hajek T Slaney CM Garnham JS . Insulin resistance and outcome in bipolar disorder. Br J Psychiatry. (2015) 206:52–7. doi: 10.1192/bjp.bp.114.152850, 25323142

[30] Phelps JR Siemers SV El-Mallakh RS. The ketogenic diet for type II bipolar disorder. Neurocase. (2013) 19:423–6. doi: 10.1080/13554794.2012.690421, 23030231

[31] Chmiel I. Ketogenic diet in therapy of bipolar affective disorder—case report and literature review. Psychiatr Pol. (2022) 56:1. doi: 10.12740/PP/OnlineFirst/136356 37098202

[32] Saraga M Misson N Cattani E. Ketogenic diet in bipolar disorder. Bipolar Disord. (2020) 22:765–5. doi: 10.1111/bdi.13013, 33021024

[33] Needham N Campbell IH Grossi H Kamenska I Rigby BP Simpson SA . Pilot study of a ketogenic diet in bipolar disorder. BJPsych Open. (2023) 9:e176. doi: 10.1192/bjo.2023.568, 37814952 PMC10594182

[34] Danan A Westman EC Saslow LR Ede G. The ketogenic diet for refractory mental illness: a retrospective analysis of 31 inpatients. Front Psychol. (2022) 13:951376. doi: 10.3389/fpsyt.2022.951376, 35873236 PMC9299263

[35] Sethi S Wakeham D Ketter T Hooshmand F Bjorstead J Richards B . Ketogenic diet intervention on metabolic and psychiatric health in bipolar and schizophrenia: a pilot trial. Psychiatry Res. (2024) 335:115866. doi: 10.1016/j.psychres.2024.115866, 38547601

[36] Chouinard V.-G. Öngür D. (n.d.). Ketogenic and nutritional interventions for first episode bipolar disorder. ClinicalTrials.gov ID: NCT06221852.

[37] Ford J.. (n.d.). Can neural network instability in schizophrenia be improved with a very low carbohydrate ketogenic diet? ClinicalTrials.gov ID: NCT05268809.

[38] Miklowitz D. J.. (n.d.). Child bipolar network ketogenic diet approach to bipolar disorder in adolescents (CBN Keto). ClinicalTrials.gov ID: NCT06920940.

[39] Ruusunen A.. (n.d.). Ketogenic diet for psychotic disorders (PsyDiet). ClinicalTrials.gov ID: NCT03873922.

[40] Kelly D.. (n.d.). Ketogenic diet in people with schizophrenia. ClinicalTrials.gov ID: NCT05968638.

[41] Longhitano C. Sarnyai Z.. (n.d.). The effects of diet on metabolic and mental health outcome measures in bipolar disorder and schizophrenia: a randomized controlled clinical trial. ANZCTR trial ID: ACTRN12623000854639.

[42] Brietzke E. M (n.d.). Ketogenic diet therapy major depressive disorder (KETOMDD). ClinicalTrials.gov ID: NCT05558995.

[43] Frank G.. (n.d.). Therapeutic ketogenic diet in anorexia nervosa. ClinicalTrials.gov ID: NCT06000774.

[44] Phillips M.. (n.d.). Examining neurobiological mechanisms underlying the therapeutic effect of the ketogenic diet in bipolar disorder (BD). ClinicalTrials.gov ID: NCT06081426.

[45] Liwinski T.. (n.d.). Ketogenic diet for depression (KETO-MOOD). ClinicalTrials.gov ID: NCT06105762.

[46] Sethi S.. (n.d.). Ketogenic diet intervention in schizophrenia, bipolar disorder, major depressive disorder: deep omic profiling. ClinicalTrials.gov ID: NCT06748950.

[47] Patikorn C Saidoung P Pham T Phisalprapa P Lee YY Varady KA . Effects of ketogenic diet on health outcomes: an umbrella review of meta-analyses of randomized clinical trials. BMC Med. (2023) 21:196. doi: 10.1186/s12916-023-02874-y, 37231411 PMC10210275

[48] Goldberg IJ Ibrahim N Bredefeld C Foo S Lim V Gutman D . Ketogenic diets, not for everyone. J Clin Lipidol. 15:61–7. doi: 10.1016/j.jacl.2020.10.005, 33191194 PMC7887024

[49] Aas IM. Guidelines for rating global assessment of functioning (GAF). Ann General Psychiatry. (2011) 10:2–11. doi: 10.1186/1744-859X-10-2, 21251305 PMC3036670

[50] Spearing MK Post RM Leverich GS Brandt D Nolen W. Modification of the clinical global impressions (CGI) scale for use in bipolar illness (BP): the CGI-BP. Psychiatry Res. (1997) 73:159–71. doi: 10.1016/S0165-1781(97)00123-6, 9481807

[51] Kroenke K Spitzer RL Williams JB. The PHQ-9: validity of a brief depression severity measure. J Gen Intern Med. (2001) 16:606–13. doi: 10.1046/j.1525-1497.2001.016009606.x, 11556941 PMC1495268

[52] Kühner C Bürger C Keller F Hautzinger M. Reliability and validity of the revised Beck depression inventory (BDI-II) results from German samples. Nervenarzt. (2007) 78:651–6. doi: 10.1007/s00115-006-2098-7, 16832698

[53] Trajković G Starčević V Latas M Leštarević M Ille T Bukumirić Z . Reliability of the Hamilton rating scale for depression: a meta-analysis over a period of 49 years. Psychiatry Res. (2011) 189:1–9. doi: 10.1016/j.psychres.2010.12.007, 21276619

[54] Zhong B Wang Y Chen H Wang X. Reliability, validity and sensitivity of Montgomery-asberg depression rating scale for patients with current major depression disorder. Chin J Behav Med Brain Sci. (2011):85–7.

[55] Mansoor N Vinknes KJ Veierød MB Retterstøl K. Effects of low-carbohydrate diets v. low-fat diets on body weight and cardiovascular risk factors: a meta-analysis of randomised controlled trials. Br J Nutr. (2016) 115:466–79. doi: 10.1017/S0007114515004699, 26768850

[56] Surma S Sahebkar A Banach M. endorsed by the International Lipid Expert Panel (ILEP), Low carbohydrate/ketogenic diet in the optimization of lipoprotein(a) levels: do we have sufficient evidence for any recommendation? Eur Heart J. (2023) 44:4904–6. doi: 10.1093/eurheartj/ehad635, 37769437

[57] Mach F Baigent C Catapano AL Koskinas KC Casula M Badimon L . 2019 ESC/EAS Guidelines for the management of dyslipidaemias: lipid modification to reduce cardiovascular risk. Eur Heart J. (2020) 41:111–88. doi: 10.1093/eurheartj/ehz455, 31504418

[58] Lamarche B Lemieux I Després JP. The small, dense LDL phenotype and the risk of coronary heart disease: epidemiology, patho-physiology and therapeutic aspects. Diabetes Metab. (1999) 25:199–211. 10499189

[59] Hoogeveen RC Gaubatz JW Sun W Dodge RC Crosby JR Jiang J . Small dense low-density lipoprotein-cholesterol concentrations predict risk for coronary heart disease: the Atherosclerosis Risk In Communities (ARIC) study. Arterioscler Thromb Vasc Biol. (2014) 34:1069–77. doi: 10.1161/ATVBAHA.114.303284, 24558110 PMC3999643

[60] St-Pierre AC Cantin B Dagenais GR Mauriege P Bernard PM Despres JP . Low-density lipoprotein subfractions and the long-term risk of ischemic heart disease in men: 13-year follow-up data from the Quebec Cardiovascular Study. Arterioscler Thromb Vasc Biol. (2005) 25:553–9. doi: 10.1161/01.ATV.0000154144.73236.f4, 15618542

[61] Tsai MY Steffen BT Guan W McClelland RL Warnick R McConnell J . New automated assay of small dense low-density lipoprotein cholesterol identifies risk of coronary heart disease: the multi-ethnic study of atherosclerosis. Arterioscler Thromb Vasc Biol. (2014) 34:196–201. doi: 10.1161/ATVBAHA.113.302401, 24233487 PMC4211254

[62] Ai M Otokozawa S Asztalos BF Ito Y Nakajima K White CC . Small dense LDL cholesterol and coronary heart disease: results from the framingham offspring study. Clin Chem. (2010) 56:967–76. doi: 10.1373/clinchem.2009.137489, 20431054 PMC3791882

[63] Mora S Szklo M Otvos JD Greenland P Psaty BM Goff DC Jr . LDL particle subclasses, LDL particle size, and carotid atherosclerosis in the Multi-Ethnic Study of Atherosclerosis (MESA). Atherosclerosis. (2007) 192:211–7. doi: 10.1016/j.atherosclerosis.2006.05.007, 16765964

[64] Sacks FM Campos H. Low-Density Lipoprotein Size and Cardiovascular Disease: A Reappraisal. J Clin Endocrinol Metab. (2003) 88:4525–32. doi: 10.1210/jc.2003-030636, 14557416

[65] Sniderman AD Thanassoulis G Glavinovic T Navar AM Pencina M Catapano A . Apolipoprotein B particles and cardiovascular disease: a narrative review. JAMA Cardiol. (2019) 4:1287–95. doi: 10.1001/jamacardio.2019.3780, 31642874 PMC7369156

[66] Mills EL Kelly B O‘Neill LAJ. Mitochondria are the powerhouses of immunity. Nat Immunol. (2017) 18:488–98. doi: 10.1038/ni.3704, 28418387

[67] Ji J Fotros D Sohouli MH Velu P Fatahi S Liu Y. The effect of a ketogenic diet on inflammation-related markers: a systematic review and meta-analysis of randomized controlled trials. Nutr Rev. (2024) 83:40–58. doi: 10.1093/nutrit/nuad175, 38219223

[68] Veech RL. The determination of the redox states and phosphorylation potential in living tissues and their relationship to metabolic control of disease phenotypes. Biochem Mol Biol Educ. (2006) 34:168–79. doi: 10.1002/bmb.2006.49403403168, 21638666

[69] Amaki T Suzuki T Nakamura F Hayashi D Imai Y Morita H . Circulating malondialdehyde modified LDL is a biochemical risk marker for coronary artery disease. Heart. (2004) 90:1211–3. doi: 10.1136/hrt.2003.018226, 15367526 PMC1768480

[70] Pérez-Torres I Manzano-Pech L Rubio-Ruíz ME Soto ME Guarner-Lans V. Nitrosative stress and its association with cardiometabolic disorders. Molecules. (2020) 25:2555. doi: 10.3390/molecules25112555, 32486343 PMC7321091

