# Correction: Case series: Effects of a ketogenic diet on cardiometabolic health in seven outpatients with bipolar disorder

**DOI:** 10.3389/fnut.2025.1750745

**Published:** 2026-01-07

**Authors:** Louis Schreel, Maxi Bürkle, Andrea Thiem, Volker von Baehr, Joshua Sauren, Gerrit Keferstein

**Affiliations:** 1 MOJO Institut für Regenerationsmedizin, Hennef, Germany; 2 IMD Labor, Berlin, Germany

**Keywords:** metabolic psychiatry, ketogenic metabolic therapy, bipolar disorder, cardiometabolic health, lipids-metabolism, inflammation, oxidative stress

The authors Andrea Thiem and Volker von Baehr were omitted as authors in the published article. The correct author list should read as:

Louis Schreel^1*^, Maxi Bürkle^1^, Andrea Thiem^2^, Volker von Baehr^2^, Joshua Sauren^1^, Gerrit Keferstein^1^

The Author Contributions Statement has been corrected to include the contributions of the added authors, to read as the following:

LS: Writing – original draft, Formal analysis, Writing – review & editing, Project administration, Funding acquisition, Visualization, Methodology, Supervision, Software, Investigation, Resources, Conceptualization, Data curation, Validation. MB: Validation, Investigation, Conceptualization, Resources, Funding acquisition, Supervision, Writing – review & editing, Software, Writing – original draft, Formal analysis, Methodology, Visualization, Data curation, Project administration. AT: Validation, Investigation, Conceptualization, Resources, Funding acquisition, Supervision, Writing – review & editing, Software, Writing – original draft, Formal analysis, Methodology, Visualization, Data curation, Project administration. VB: Validation, Investigation, Conceptualization, Resources, Funding acquisition, Supervision, Writing – review & editing, Software, Writing – original draft, Formal analysis, Methodology, Visualization, Data curation, Project administration. JS: Software, Investigation, Visualization, Supervision, Funding acquisition, Conceptualization, Writing – review & editing, Formal analysis, Project administration, Resources, Data curation, Writing – original draft, Methodology, Validation. GK: Supervision, Writing – review & editing, Methodology, Software, Conceptualization, Writing – original draft, Investigation, Funding acquisition, Visualization, Formal analysis, Validation, Resources, Project administration, Data curation.

The affiliation IMD Labor Berlin was omitted from the article as published. This affiliation has now been added for author(s) [Andrea Thiem, Volker von Baehr].

The original version of this article has been updated.

