# Supplementary material for: Case series: Effects of a ketogenic diet on cardiometabolic health in seven outpatients with bipolar disorder
Source: Front Nutr. 2025 Oct 7;12:1635489. doi: 10.3389/fnut.2025.1635489 (PMC12538888; doi:10.3389/fnut.2025.1635489)
Supplement: Supplementary file 1 [file Data_Sheet_1.pdf]

Supplementary Data: Ketogenic Metabolic Therapy Intervention

Clinical Setting

Ketogenic metabolic therapy was provided through an outpatient medical practice consisting of a medical doctor experienced in ketogenic metabolic therapies, a psychologist experienced in psychiatric care, and an intern ketogenic dietitian-nutritionist, under the supervision of the treating medical doctor. All clinical assessment, education, intervention, community and lifestyle support was provided by licensed medical staff, either in-person or virtually.

Inclusion and exclusion criteria

Eligible participants had to have a diagnosis of bipolar disorder type 1 or 2 and had to be clinically euthymic for at least three months (i.e. have had no episodes of depression or hypomania/mania). They had to be between the age of 18 and 70 and needed to have a sufficient understanding of the German language and live in Germany. Further exclusion criteria were: pregnancy, breastfeeding or planning to become pregnant within the next 3 months; active substance misuse; prior use of KD within 2 months; following a vegan or vegetarian diet; admission to hospital within 3 months; involvement in any research; inability to complete baseline assessments; liver, kidney or cardiovascular disease, severe hyperlipidemia; and disorders of fat/ketone metabolism. Patients taking SGLT2 inhibitors could not participate in the program due to the risk of ketoacidosis. Written informed consent was obtained from all participants to pursue KMT and to allow publications of de-identified data.

Comprehensive Clinical Evaluation

Seven adult outpatients, ages 29 – 50, with bipolar disorder types 1 and 2, were selected for admission to our KetoBrain program. Four patients were on anti-epileptics, three on anti-depressants, two on lithium and two on antipsychotics.

Case 1: Lithium 450 mg 1-0-2; Bupropion 300mg 1-0-0; Metformin 1000mg 1-0-1
Case 2: Sertraline 50mg 1-0-0; Lamotrigine 200 mg 1-0-1; Quetiapine 50mg 0-0-1-2
Case 3: No medications
Case 4: Lamotrogin 50mg 1-0-1; Escitalopram 15mg
Case 5: Lithium 450 mg 1-0-1.5; Quetiapine 150 mg 0-0-1
Patient 6: Valproate 1000mg 1-0-1; Sertraline 200mg 1-0-0
Patient 7: Quetiapine 275mg 0-0-1, Valproate 200mg 0-0-1

The medical history revealed notable comorbidities in the cohort. All patients met the formal diagnostic criteria for dyslipidemia prior to the onset of treatment, characterized by elevated triglycerides, reduced HDL cholesterol, or elevated LDL cholesterol. One

patient also met all diagnostic criteria for metabolic syndrome, including abdominal obesity, elevated triglycerides, reduced HDL cholesterol, elevated blood pressure, and elevated fasting glucose. Additional medical conditions included ankylosing spondylitis (Morbus Bechterew) and recurrent migraines in one patient, prediabetes in another, and irritable bowel syndrome (IBS) in a third. Another patient presented with a combination of sleep apnea, chronic pain syndrome, and tinnitus. Psychiatric comorbidities were observed in two cases: one patient was diagnosed with generalized anxiety disorder and ADHD alongside bipolar disorder. Another patient's history was remarkable for a severe case of Lyme disease immediately preceding the onset of their first depressive episode at the age of 18, with a formal diagnosis of bipolar disorder following three years later. This temporal relationship between Lyme disease and mood symptoms raises questions about potential triggering or contributing mechanisms. Patients underwent a comprehensive medical evaluation, psychiatric evaluation, detailed nutritional assessment, and lifestyle assessment. A day-long education session was offered to discuss treatment goals, risks and benefits of ketogenic dietary therapy, and the proper implementation of ketogenic dietary therapy.

Medical Screening and Initial Assessments

Medical records and recent laboratory results were reviewed to verify the patients' medical histories and to anamnestically rule out conditions that contraindicate ketogenic metabolic therapy (KMT). These included pancreatitis, severe liver disease, pyruvate carboxylase deficiency, fatty acid oxidation defects, primary carnitine deficiency, palmitoyl transferase I or II deficiency, carnitine translocase deficiency, and acute intermittent porphyria. Additionally, the review aimed to identify medical conditions requiring attention before and during KMT.

Comprehensive screening laboratories were conducted, encompassing metabolic and organ function tests such as a comprehensive metabolic profile, liver and kidney function assessments, lipid and inflammation markers, and specialized profiles. These included measurements of carnitine, urea, and electrolyte levels, HbA1c, as well as comprehensive microbiome, micronutrient, and amino acid metabolism profiles. Further evaluations included omega-3, omega-6, mono-unsaturated, and saturated fatty acid profiles, along with short-chain fatty acids in stool and serum. Additional clinical assessments involved heart rate variability, bioelectrical impedance analysis (BIA) for body composition, and blood pressure measurements.

From the outset, patients engaged in detailed and frequent discussions about the risks and benefits of KMT. These conversations addressed urgency measures for managing hypoglycemia, hypotension, and elevated serum beta-hydroxybutyrate (BHB) levels above 6.0 mmol/L, as well as the potential challenges of sleep deprivation and signs of

hypomania. Emphasis was placed on the importance of close psychiatric and medical monitoring through both in-person and virtual consultations, the use of supportive medication in cases of sleep deprivation or hypomania, and the necessity of daily ketone testing using the Biocoach blood ketone meter to monitor BHB levels. This comprehensive preparation ensured that all medical, psychiatric, and safety considerations were thoroughly addressed prior to initiating KMT.

Ketogenic dietary therapy implementation

Before initiating the ketogenic diet, a transition period of two weeks was prescribed, which consisted of a whole foods dietary pattern, essentially eliminating all grains, legumes and products with added sugar. This paleo-style diet consisted of meat, seafood, poultry, eggs, dairy, fruits and vegetables, and nuts and seeds, without any specific restrictions in terms of macronutrient ratio. In this period, a well-formulated, personalized ketogenic diet was developed for each patient by an intern ketogenic dietitian, under supervision of the treating medical doctor. The following principles were followed. First, ketogenic macronutrient ratios ranged from 1.5:1 to 2.0:1 (fat: protein + carbohydrates), with macronutrient ranges of approximately 75% fat, 20% protein, 5% or less carbohydrate. Second, patients were asked to eat no more than 20g of carbohydrates per day – counting so-called "net carbohydrates" as "total carbohydrates" minus fiber. Third, patients were asked to consume 50% of total fat intake from mono-unsaturated fats, 30% from poly-unsaturated fats and 20% from saturated fats. Fourth, patients were asked to prioritize fiber intake through vegetables and seeds low in carbohydrates. Based on these principles and personal preferences, a personalized KMT plan was provided with standard recipes and meal plans.

After the first two weeks, the personalized KMT plan was implemented, starting a two-week period of keto-adaptation. Several principles guided this period. First, patients were asked to measure blood ketone levels 3 times per day: once 60 minutes after waking still before breakfast, once two hours after lunch and once two hours after diner. Patients were asked to aim for blood BHB levels of between 1.0 mmol/L and 5 mmol/L. Second, patients were asked to closely monitor sleep and possible signs of oncoming hypomania, and to report these to their treating medical doctor and psychiatrist. Third, in line with international recommendations, patients were encouraged to drink more fluids and to adopt daily mineral supplementation consisting of sodium (5g of sodium chloride per day), potassium (3g as potassium chloride or potassium citrate per day), and magnesium (400 mg of magnesium malate or magnesium glycinate per day). Fourth, all participants were asked to record their food intake in an online food diary (e.g. Cronometer), which was reviewed regularly. Accordingly, participants were asked to weigh all dietary foods with a scale for at least the first 2 weeks of the study. As soon as the target blood ketone levels were safely and structurally achieved, weighing the food and maintaining the food diary was no longer necessary.

Standardized Therapeutic Micronutrients Protocol

Each KMT followed a standardized micronutrient supplementation protocol as detailed below. First, when bioactive vitamin B6 levels were below 10.1, 25 mg of vitamin B6 was prescribed as P-5-P for 90 days. Second, if bioactive vitamin B12 levels were below 300 and vitamin B9 levels below 90, 1 mg of vitamin B12 and 0.4 mg of vitamin B9 (as L-5-methyl-folate) were prescribed daily in a single tablet. Third, if bioactive vitamin B9 levels were below 90, 400 µg of folate was prescribed daily. Fourth, if vitamin D levels were below 50, patients were advised to spend 30 minutes in the sun daily or use a solarium twice per week.

For omega-3, when the omega-3 index was below 5, 3 capsules of 1800 mg omega-3 were prescribed daily, and when the omega-3 index was below 8 but above 5, 1 capsule of 600 mg omega-3 was prescribed daily. In cases of general amino acid deficiency, patients were instructed to take 1 capsule of digestive enzymes before large meals or, alternatively, consume a shot of apple cider vinegar 10 minutes before eating, practice mindful eating, or include raw eggs in their diet. If selenium levels were below 90, 200 µg of selenium was prescribed daily for 30 days. Lastly, if ferritin levels were below 30, patients received an iron infusion.

This comprehensive protocol ensured that micronutrient deficiencies were addressed systematically and effectively to support optimal outcomes during KMT.

Standardized Therapeutic Exercise Protocol

Participants expressed interest in understanding how to incorporate exercise into their ketogenic metabolic therapy (KMT). To address this, we provided a standardized exercise protocol tailored to individual fitness levels and goals.

The primary focus was on maximizing low-intensity exercise, such as walking, leisurely cycling, or light hiking. For beginners, the target was set at 6 hours of low-intensity exercise per week. Intermediate participants were encouraged to aim for 10 hours per week, while advanced participants had a target of up to 16 hours per week.

For higher-intensity activities, recommendations varied based on experience. Beginners were advised to engage in up to four sessions of 30 minutes per week, focusing on bodyweight exercises, functional training, or light recreational sports. Intermediate and advanced participants were encouraged to perform up to four sessions of 60 minutes per week, incorporating strength training or more intensive sports.

This protocol was designed to enhance metabolic flexibility, optimize physical well-being, and support overall adherence to the KMT regimen while accommodating individual capabilities and progress.
